# An unconventional SNARE complex mediates exocytosis at the plasma membrane and vesicular fusion at the apical annuli in *Toxoplasma gondii*

**DOI:** 10.1371/journal.ppat.1011288

**Published:** 2023-03-27

**Authors:** Jiawen Fu, Lin Zhao, Juan Yang, Heming Chen, Shinuo Cao, Honglin Jia

**Affiliations:** State Key Laboratory for Animal Disease Control and Prevention, Harbin Veterinary Research Institute, Chinese Academy of Agricultural Sciences (CAAS), Harbin, China; University of Geneva, SWITZERLAND

## Abstract

Exocytosis is a key active process in cells by which proteins are released in bulk via the fusion of exocytic vesicles with the plasma membrane. Soluble N-ethylmaleimide-sensitive factor attachment protein receptor (SNARE) protein-mediated vesicle fusion with the plasma membrane is essential in most exocytotic pathways. In mammalian cells, the vesicular fusion step of exocytosis is normally mediated by Syntaxin-1 (Stx1) and SNAP25 family proteins (SNAP25 and SNAP23). However, in *Toxoplasma gondii*, a model organism of Apicomplexa, the only SNAP25 family protein, with a SNAP29-like molecular structure, is involved in vesicular fusion at the apicoplast. Here, we reveal that an unconventional SNARE complex comprising TgStx1, TgStx20, and TgStx21 mediates vesicular fusion at the plasma membrane. This complex is essential for the exocytosis of surface proteins and vesicular fusion at the apical annuli in *T*. *gondii*.

## Author summary

Soluble N-ethylmaleimide-sensitive factor attachment protein receptor (SNARE) proteins that mediate exocytosis in *T*. *gondii* have not been well identified. In this study, the functions of two unique SNAREs, TgStx20 and TgStx21, that share homology with the C- and N-terminal SNARE domains of SNAP23 were investigated through tagging an auxin-inducible degron system. The results indicate that both SNAREs are required for the lytic cycle of this parasite. Depletion of TgStx20 or TgStx21 caused a deficiency of exocytosis of surface proteins. In particular, the SNARE proteins located in *T*. *gondii* apical annuli were demonstrated to be required for the fusion of Rab11A-positive vesicles at these sites for the first time. We also found that a conserved Qa SNARE, TgStx1, forms a complex with TgStx20 and TgStx21. Depletion of TgStx1 in parasites caused similar phenotypes as those observed in TgStx20- or TgStx21-deficient parasites. These data reveal a unique SNARE complex that mediates vesicular fusion at the plasma membrane and provide further insight into the molecular mechanism of the vesicular trafficking pathway to the apical annuli.

## Introduction

*Toxoplasma gondii* is an obligate intracellular parasitic protozoan with a complicated life cycle that can infect almost all warm-blooded animals [1]. Infections of *T*. *gondii* in healthy individuals are typically asymptomatic, and the parasites are maintained in host cells as chronic infections of tissue cysts. However, in immunodeficient patients, the latent infection can be reactivated and may lead to the development of life-threatening encephalitis [2]. Infection in pregnant women may result in abortion or vertical transmission to the fetus, causing severe pathologic defects [3].

A fundamental hallmark of eukaryotic cells is their segregation into functionally distinct organelles, including those of the secretory and endocytic pathways [4]. Vesicular transport is thus a critical cellular activity responsible for trafficking between various membrane-enclosed compartments. Transport and exocytosis of secreted proteins also rely on this vesicular trafficking. Proteins that traffic through the secretory system are first synthesized in the endoplasmic reticulum (ER), processed in the Golgi apparatus, and then delivered to the plasma membrane, where they are secreted or integrated into the cell membrane [5]. In both regulated and default secretion, the exocytic event terminates with the fusion of cargo-containing vesicles with the plasma membrane.

Membrane fusion between vesicles and their target regions mediated by soluble N-ethylmaleimide-sensitive factor attachment protein receptor (SNARE) proteins is a critical step for cargo transportation [6]. SNARE proteins can be categorized as Qa, Qb, Qc, or R SNAREs, depending on their position in the SNARE complex [7]. SNAP25 family proteins (including SNAP23, SNAP25, and SNAP29) contain both Qb and Qc SNARE motifs. The function of SNAP25 and SNAP23 in regulated and default secretion has been extensively documented [8–12]. By contrast, SNAP29, together with syntaxin-17 (a Qa SNARE) and VAMP8 (an R SNARE), are required for the fusion of autophagosomes with endosomes/lysosomes in mammalian cells, resulting in the generation of degradative autolysosomes [13].

In *T*. *gondii*, only one Qbc SNARE protein (TgSNAP29) has been identified. This protein lacks clear palmitoylation sites in the junction of the two SNARE structural domains, which play a role in apicoplast biogenesis in parasites [14]. There are no clear orthologs of SNAP25 and SNAP23 in *T*. *gondii*. A recent study reported that TgRab11A is involved in the secretion of dense granule proteins (GRAs) and microneme proteins (MICs) and the transport of transmembrane proteins. During the invasion of host cells, TgRab11A aggregates with GRA proteins at the apical region of parasites, indicating it participates in the secretion of proteins at the early stage of invasion [15]. However, no SNARE complex that mediates vesicular fusion for exocytosis at the plasma membrane has been found in *T*. *gondii*. It is unknown how the membrane fusion step in the exocytotic process is executed in this parasite.

In our previous study, we identified 26 SNARE proteins in *T*. *gondii*. Here, we report that an unconventional SNARE complex comprising TgStx1, TgStx20, and TgStx21 is located at the surface of the parasite membrane and the apical annuli. Depletion of TgStx1, TgStx20, or TgStx21 in these parasites causes marked defects in the exocytosis of surface proteins and leads to a deficiency of vesicular fusion at the apical annuli.

## Results

### The genome of *T*. *gondii* encodes two unique SNARE proteins that share homology with SNAP23

**Fig 1:**
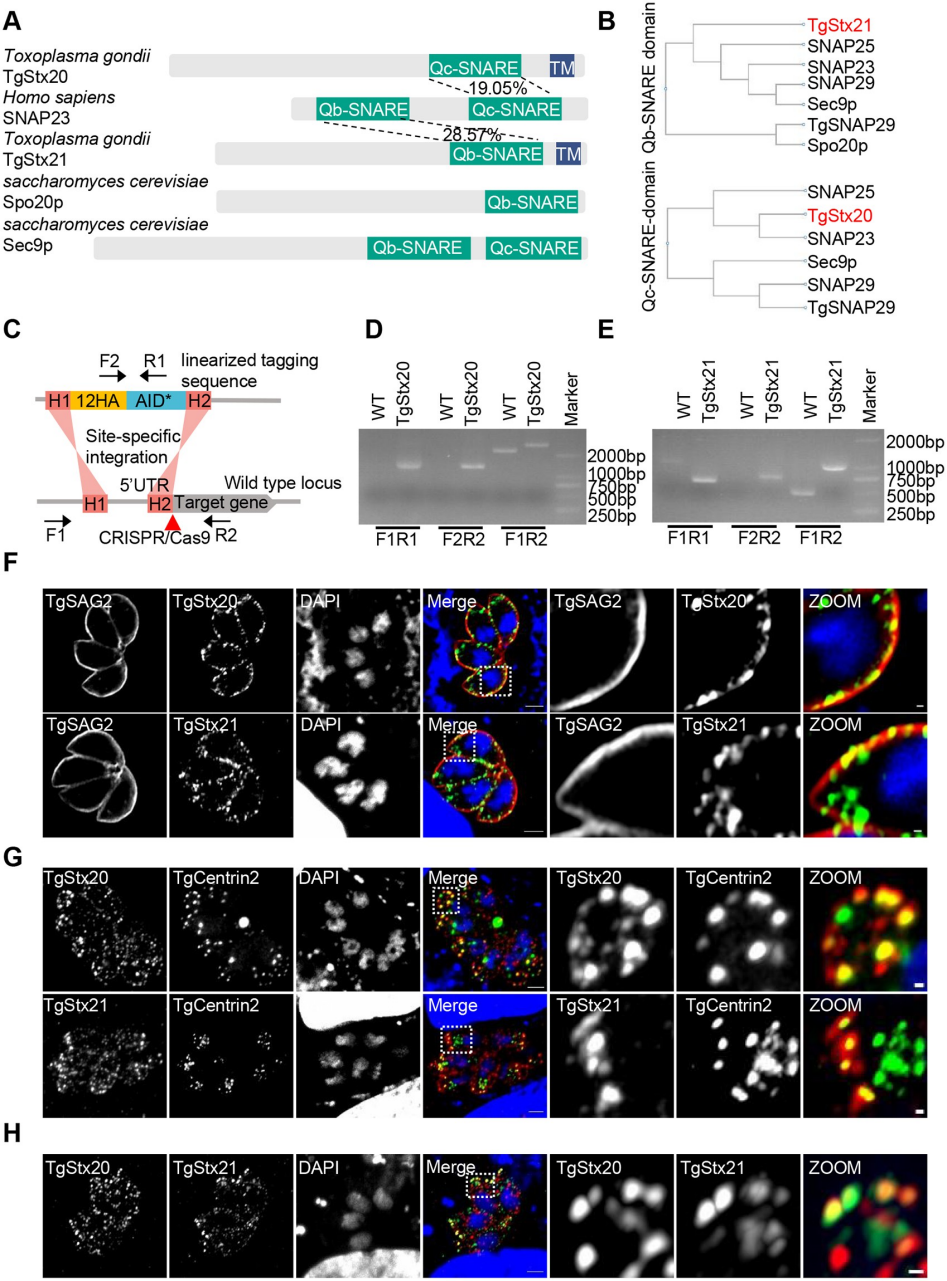
TgStx20 and TgStx21 localize to the plasma membrane and apical annuli. (**A**) The molecular architecture of SNAP25 family proteins. The percent identity values are shown. (**B**) Phylogenetic analysis of Apicomplexan, *Saccharomyces cerevisiae*, and human SNAP25 family proteins and homologs. (**C**) Schematic representation of the AID*-based system used to conditionally deplete the SNAREs. (**D**) and (**E**) PCR analysis of the integration of 12HA-AID*-TgStx20 (D) and 12HA-AID*-TgStx21 (E) at the genomic locus using the indicated primers. (**F**) IFA showing that 12HA-AID*-TgStx20 (green) and 12HA-AID*-TgStx21 (green) partially colocalized with the plasma membrane protein TgSAG2 (red). Scale bars: 2 μm (left panels) and 0.2 μm (zoomed panels). (**G**) IFA showing that TgStx20 (red) and TgStx21 (red) partially colocalized with the apical annuli protein TgCentrin2 (green). 12HA-AID*-TgStx20 or 12HA-AID*-TgStx21 parasites were transiently transfected with a plasmid expressing TgCentrin2 fused with EGFP and subjected to an IFA. Scale bars: 2 μm (left panels) and 0.2 μm (zoomed panels). (**H**) IFA showing that TgStx20 (red) completely colocalized with TgStx21 (green) at the apical annuli. A 4MYC tag was inserted at the N-terminus of endogenous TgStx20 in 12HA-AID*-TgStx21 parasites. Scale bars: 2 μm (left panels) and 0.2 μm (zoomed panels).

In our previous study, the subcellular localizations of SNAREs were analyzed in *T*. *gondii*. Two unique SNAREs seem to accumulate at the apical region of parasites. These two SNAREs, which were named as TgStx20 (TGGT1_253360) and TgStx21 (TGGT1_306640), share homology with the C- and N-terminal SNARE domains of SNAP23 but clearly contain a transmembrane domain (Fig 1A). By comparing the SNARE domains of these SNAREs with SNAP25 family proteins in human and yeast (Fig 1B), we found that the SNARE domain of TgStx20 is more similar to the exocytic SNAREs (SNAP25 and SNAP23) of human, but the SNARE domain of TgStx21 is more distinct with other Qb SNARE domains of SNAP family proteins. These data suggest that TgStx20 and TgStx21 evolved independently in parasites. We then constructed two conditional knockout strains of TgStx20 and TgStx21 using the AID conditional knockout system (Fig 1C). Diagnostic PCR analyses and sequencing confirmed the correct integration. 12HA-AID* successfully fused with the N-termini of TgStx20 and TgStx21 (Fig 1D and 1E). The localizations of these two proteins were investigated by an indirect immunofluorescence assay (IFA). The signals of TgStx20 and TgStx21 appeared as dispersed puncta in the cytoplasm and clustered in the plasma membrane (Fig 1F). SNAREs are concentrated in clusters that define docking and fusion sites for exocytosis [16,17]. The punctate staining of TgStx20 and TgStx21 might represent the exocytic sites of secretory vesicles at the plasma membrane.

Interestingly, in addition to their plasma membrane localization, TgStx20 and TgStx21 also clearly clustered in the apical region of parasites, showing regular symmetrical puncta, similar to the localization of a unique structure of parasites called the apical annuli [18]. Co-localization analysis of TgStx20 and TgStx21 with Tgcentrin2 confirmed their localization at these sites (Fig 1G). Co-localization of TgStx20 and TgStx21 in the apical annuli was confirmed by insertion of 4MYC at the N-terminus of endogenous TgStx21 in 12HA-AID*-TgStx20 parasites (Fig 1H).

### TgStx20 and TgStx21 are essential for the lytic cycle of *T*. *gondii*

**Fig 2:**
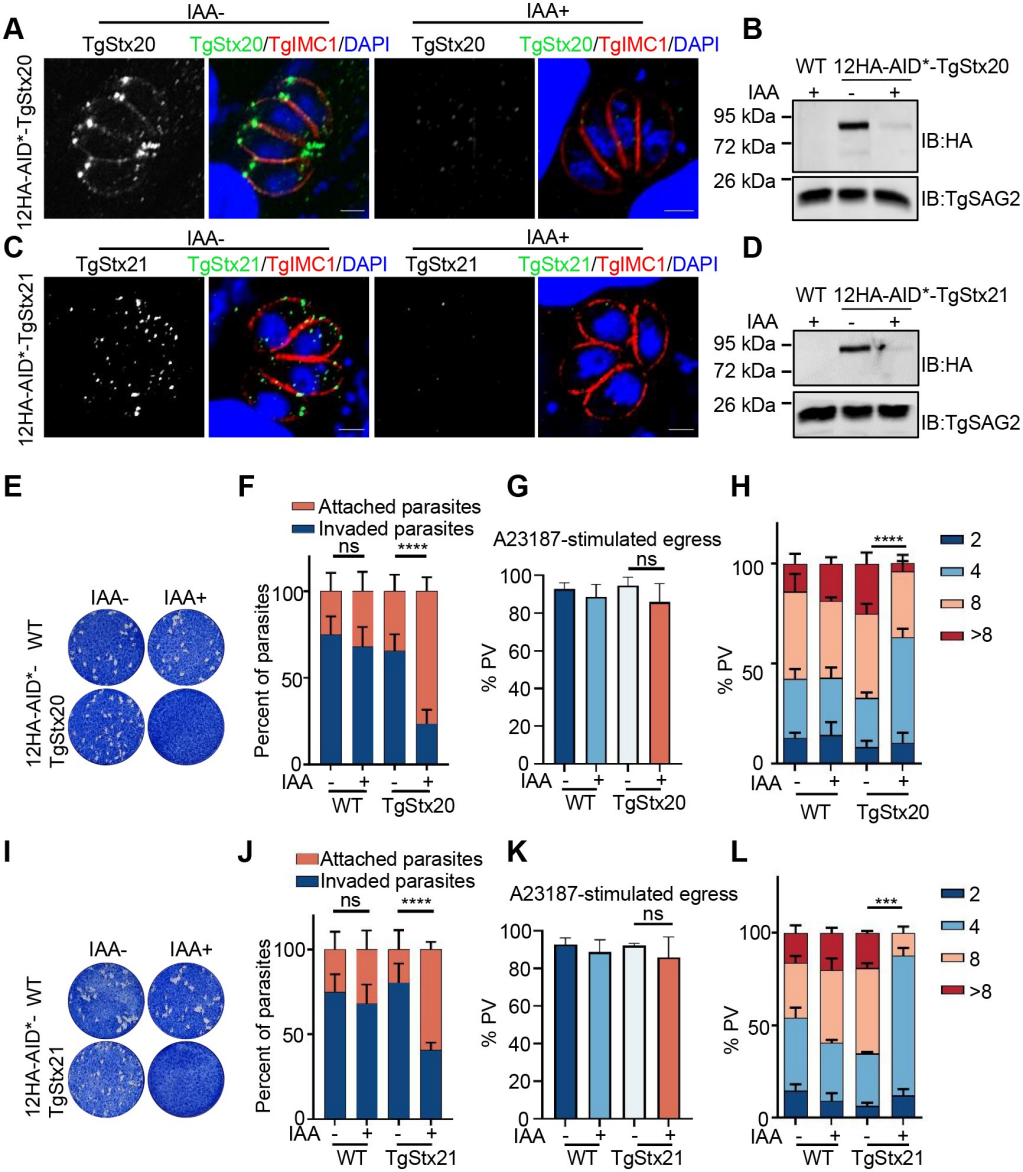
TgStx20 and TgStx21 are essential for the lytic cycle of *T*. *gondii*. (**A**) and (**C**) 12HA-AID*-TgStx20 and 12HA-AID*-TgStx21 parasites were cultured with or without IAA for 24 h and stained with antibodies against TgIMC1 (red) and HA (green). Scale bars: 2 μm. (**B**) and (**D**) The parasites were cultured in the presence of IAA for 12 h in vivo and subjected to WB analysis. (**E**) and (**I**) Plaque assays were performed by infecting BJ-5ta cells with 12HA-AID*-TgStx20 (E) and 12HA-AID*-TgStx21 (I) parasites for 8 d in the presence or absence of IAA. (**F**) and (**J**) Invasion assays examining the ability of 3HA-AID*-TgStx20 (F) and 12HA-AID*-TgStx21 (J) parasites to attach to and invade host cells in the presence or absence of IAA. Parasites were grown for 6 h in the presence or absence of IAA before egression. The data were analyzed for statistical significance by the unpaired t-test; ****P<0.0001. (**G**) and (**K**) Egress of 12HA-AID*-TgStx20 (G) and 12HA-AID*-TgStx21 (K) parasites from host cells was determined by an IFA. Parasites were grown in HFFs for 36 h and treated with IAA or vehicle (EtOH) for 8 h prior to stimulation with A23187 for 5 min. Statistical differences were analyzed by the unpaired t-test; ns. (**H**) and (**L**) Replication of 12HA-AID*-TgStx20 (H) and 12HA-AID*-TgStx21 (L) parasites after growth in the presence or absence of IAA for 24 h. The average number of parasites per vacuole was determined. The data were analyzed by a two-way ANOVA; ***P = 0.004.

Integration of an AID* tag allowed us to deplete the expression of TgStx20 and TgStx21 proteins in *T*. *gondii* by adding indole-3-acetic acid (IAA). Both 12HA-AID*-TgStx20 and 12HA-AID*-TgStx21 were effectively degraded in the presence of IAA as determined by an IFA (Fig 2A and 2C) and western blotting (WB) (Fig 2B and 2D).

To explore the effect of gene deletion on the growth of parasites, we conducted plaque assays in immortalized human foreskin fibroblast (HFF, BJ-5ta) cell monolayers with or without IAA using the AID*-tagged parasite strains and parental strain. The 12HA-AID*-TgStx20 and 12HA-AID*-TgStx21 strains did not form plaques in HFFs after 8 d of incubation with IAA but formed standard plaques in the absence of IAA (Fig 2E and 2I). To explore the role of TgStx20 and TgStx21 in the invasion of parasites, we inoculated the IAA-treated and wild-type strains into fresh HFF monolayers, rinsed out uninvaded parasites 3 h later, and counted invaded parasites within randomly selected areas to examine the invasion ability of parasites. The ability of parasites to invade host cells was significantly reduced after IAA treatment (Fig 2F and 2J). However, the egress ability of parasites was not significantly impaired (Fig 2G and 2K). In addition, proliferation in host cells was attenuated, with a low percentage of parasitophorous vacuoles (PVs) containing eight or more parasites (Fig 2H and 2L). These data indicate that a lack of TgStx20 or TgStx21 seriously affects the normal lytic cycle of these parasites.

### TgStx20 and TgStx21 are required for the exocytosis of surface proteins and proper localization of plasma transmembrane proteins

**Fig 3:**
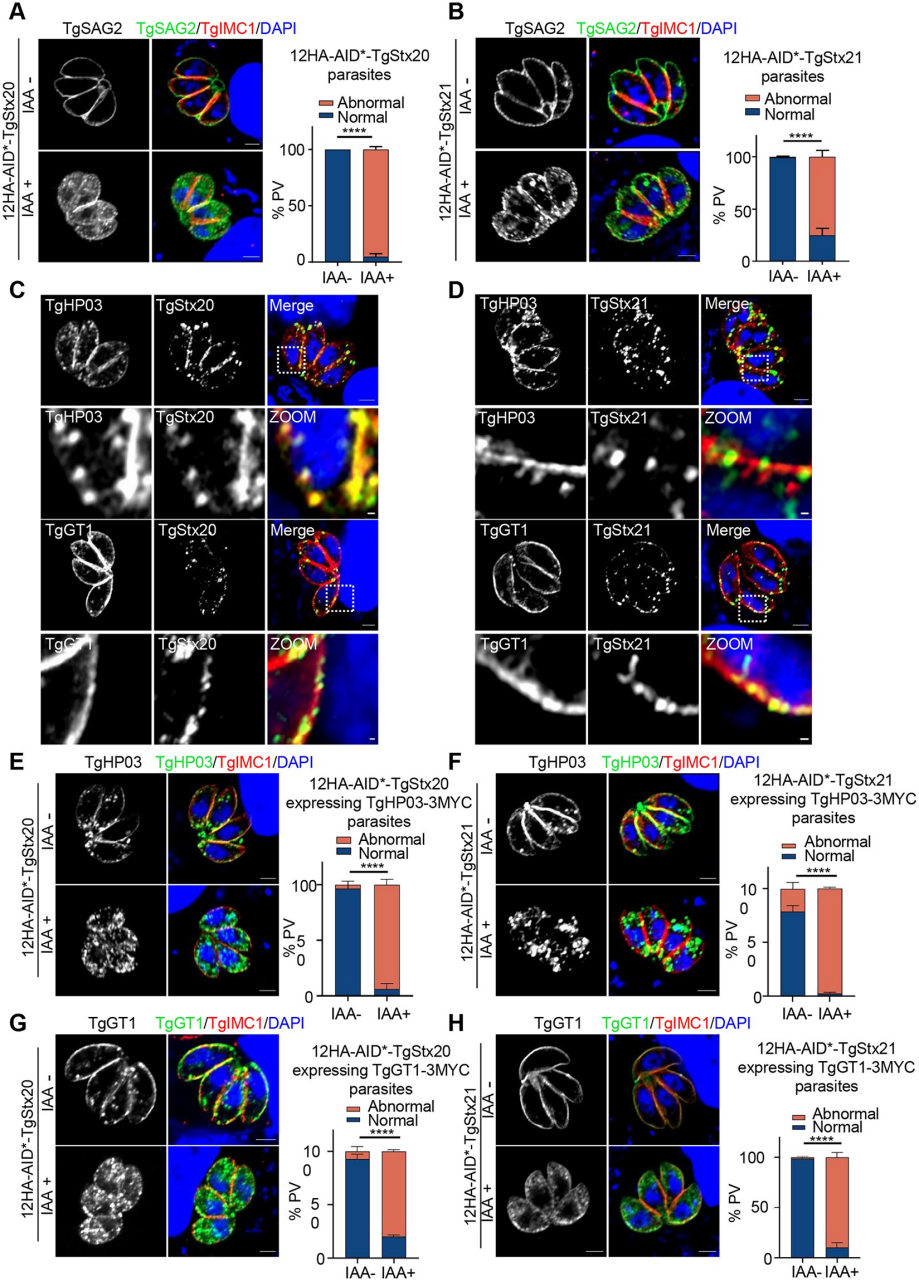
TgStx20 and TgStx21 are involved in the proper localization of transmembrane proteins to the PM. (**A**) and (**B**) IFA showing that conditional ablation of TgStx20 and TgStx21 affected the trafficking of TgSAG2 (green) to the plasma membrane. The parasite cortex was stained with a rabbit anti-TgIMC1 antibody (red). Scale bars: 2 μm. Data are mean ± SEM from three independent slides, and at least 100 PVs were counted per slide. The difference in the number of abnormal PVs was statistically analyzed by a two-way ANOVA; ****P<0.0001. (**C**) and (**D**) Co-localization of TgStx20 (green) and TgStx21 (green) with TgHP03 (red) and TgGT1 (red) using an IFA. 12HA-AID*-TgStx20 or 12HA-AID*-TgStx21 parasites were transfected with a plasmid expressing 3MYC-tagged TgHP03 or TgGT1, selected using pyrimethamine, and then analyzed by an IFA. Scale bars: 2 μm (upper panels) and 0.2 μm (zoomed panels). (**E**) and (**F**) IFA showing that TgGT1 (green) and TgHP03 (green) were retained in intra-cytosolic vesicles following treatment of 12HA-AID*-TgStx20 and 12HA-AID*-TgStx21 parasites with IAA for 24 h, but were located at the membrane without IAA treatment. The parasite cortex was stained with a rabbit anti-TgIMC1 antibody (red). Scale bars: 2 μm. Data show mean ± SEM of three independent experiments and statistical differences were analyzed by the unpaired t-test; ****P<0.0001.

Next, we analyzed the function of TgStx20 and TgStx21 in the exocytosis of surface proteins. The surface of *T*. *gondii* is coated with glycosylphosphatidylinositol (GPI)-anchored SRS (SAG1-related sequences) superfamily proteins. These proteins play essential roles in the attachment of parasites to host cells and the virulence of parasites. TgSAG2 was no longer delivered to the parasite surface and accumulated in the cytoplasm in parasites lacking TgStx20 or TgStx21 (Fig 3A and 3B). The major facilitator superfamily homolog TgHP03 is a multi-spanning ligand transmembrane transporter maintained at the parasite membrane via retromer-mediated endocytic recycling [19]. The glucose transporter TgGT1 is proficient in transporting mannose, galactose, and fructose in addition to glucose, and is a crucial hexose transporter at the parasite plasma membrane [20]. We next examined the role of TgStx20 and TgStx21 in the trafficking of these transporters to the plasma membrane by stably transfecting parasites with plasmids encoding 3MYC-tagged TgGT1 and TgHP03. The puncta of TgStx20 and TgStx21 clusters in the plasma membrane colocalized with the signals of both transporters, which might represent secretory vesicles docking at exocytic sites (Fig 3C and 3D). Depletion of TgStx20 or TgStx21 caused TgGT1 and TgHP03 to accumulate in the cytoplasm (Fig 3E and 3F. These data suggest that TgStx20 and TgStx21 might be involved in the delivery of transmembrane proteins to the plasma membrane. The localization of TgStx20 and TgStx21 was not restricted to the plasma membrane, and a large portion of TgStx20 and TgStx21 puncta was observed in the cytosol of parasites. Therefore, we also examined the proper delivery of other proteins that should traffic through the secretory pathway, including proteins destined to be delivered to the ER, Golgi apparatus, secretory organelles (MICs), and inner membrane complex (IMC). The localizations of these proteins were not affected when TgStx21 was depleted (S1A–S1D Fig). These observations suggest that exocytosis of surface proteins and proper localization of plasma transmembrane proteins are mainly interrupted at the vesicular fusion step at the plasma membrane when TgStx20 and TgStx21 are ablated in parasites.

An inability to maintain transporters at the plasma membrane probably hampers nutrient uptake by host cells. We next analyzed the metabolic activity in TgStx21-depleted parasites. In total, 17 differential metabolites were identified upon depletion of TgStx21, including 14 that displayed lower inclusion of environment-derived carbon and 3 that displayed increased inclusion (S2A Fig). Pathway analysis of these differential metabolites indicated that central carbon metabolism, including glycolysis, the pentose phosphate pathway, and galactose metabolism, were affected in TgStx21-depleted parasites, which indicates that uptake of sugar was impaired when the relevant transporters could not be delivered into the plasma membrane. Our data indicated that the glycerolipid/free fatty acid cycle was most severely impeded upon ablation of TgStx21, which might be because the salvage of lipids from the environment was hampered due to a lack of related transporters in the plasma membrane (S2B Fig). In *T*. *gondii*, normal invasion processes rely on adequate ATP produced by glycolysis and glutaminolysis [21]. The impairment of glycolysis explained the deficiency of host invasion by TgStx20- and TgStx21-depleted parasites.

The secretion of GRAs is also proposed to be a major constitutive pathway in *T*. *gondii*. Therefore, we examined the secretion of TgGRA7 in 12HA-AID*-TgStx21 parasites treated with IAA. However, we did not observe any reduction in the secretion of this protein in WB analysis (S3A and S3B Fig) and an IFA (S3C Fig). Previous studies suggest that multiple categories of dense granules might exist in *T*. *gondii* [22,23]. We further examined the exocytosis of other GRA proteins in 12HA-AID*-TgStx21 parasites using an IFA. GRA2, GRA24, and GRA52 were secreted normally when TgStx21 was depleted (S3C and S3D Fig).

### Vesicular fusion mediated by TgStx20 and TgStx21 at the apical annuli is associated with TgRab11A

Rab11, a member of the small G protein family (Rab11a, Rab11b, and Rab25), localizes to the Golgi network, post-Golgi vesicles, and circulating endosomes, and functions as a molecular switch in the endocytic and exocytic pathways [15]. Rab11a-mediated E-cadherin transport and sorting by circulating endosomes is essential for the depolarization-to-polarization transition of mammalian epithelial cells [24]. In *Trypanosoma brucei*, Rab11 assists the recycling of transmembrane proteins rather than GPI-anchored membrane proteins and plays a critical role in the balance of the endocytic and exocytic systems in the blood phase [25]. The genome of *T*. *gondii* encodes two Rab11 orthologs (TgRab11A and TgRab11B). TgRab11B mediates transport of the components of the IMC in parasites [26]. TgRab11A localizes to dynamic vesicles in the cytoplasm and is involved in the secretion of surface proteins and GRAs and the proper localization of plasma transmembrane proteins [15]. The localization of TgRab11A to the apical end of extracellular parasites is very similar to the site of apical annuli.

**Fig 4:**
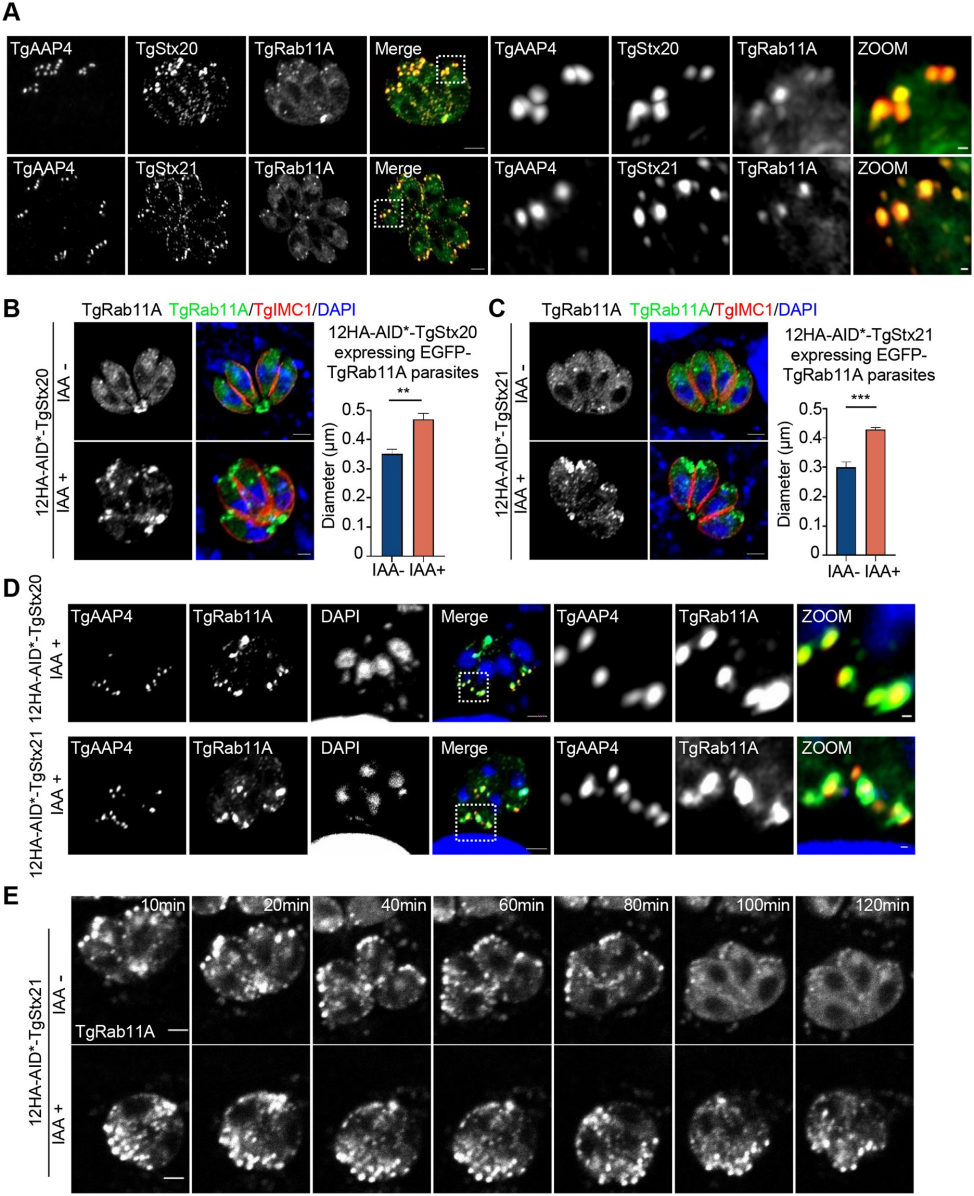
TgStx20 and TgStx21 partially localize to the apical annuli. **(A)** IFA showing that TgStx20 (orange) and TgStx21 (orange) partially colocalized with TgRab11A (green) at the apical annuli (red). A 3MYC tag was inserted at the C-terminus of endogenous TgAAP4 in 12HA-AID*-TgStx20 and 12HA-AID*-TgStx21 parasites. The parasites were transfected with a plasmid expressing FKBP-EGFP-TgRab11A, selected using pyrimethamine, and then analyzed by an IFA. Scale bars: 2 μm (left panels) and 0.2 μm (zoomed panels). **(B)** and **(C)** IFA showing that TgRab11A aggregated at the apical region following treatment of 12HA-AID*-TgStx20 and 12HA-AID*-TgStx21 parasites with IAA for 24 h, but was distributed in the cytoplasm without IAA treatment. The parasite cortex was stained with a rabbit anti-TgIMC1 antibody (red). Scale bars: 2 μm. The longest diameters of the TgRab11A puncta were measured using the distance tool of ZEN 3.3 software. One hundred Rab11A-positive puncta at the apical pole were measured in each slide. Data shown in the graphs are the mean ± SEM of three independent experiments and statistical differences were analyzed by the unpaired t-test; **P = 0.0013 and ***P = 0.0002. (**D**) IFA showing that accumulated signals of TgRab11A (green) colocalized with TgAAP4 (red) in parasites when TgStx20 or TgStx21 was depleted. The parasite cortex was stained with a rabbit anti-TgIMC1 antibody. Scale bars: 2 μm (left panels) and 0.2 μm (zoomed panels). (**E**) Sequential images extracted from S1 and S2 Movies showing that the accumulation of TgRab11A signals at the apical annuli gradually diminished after IAA was washed out. Scale bars: 2 μm.

To determine whether the vesicular fusion mediated by TgStx20 and TgStx21 at these sites is related to TgRab11A, we overexpressed TgRab11A fused with an FKBP tag and a GFP tag at the N-terminus in strains expressing AID*-tagged TgStx20 and TgStx21. In the absence of Shield-1, FKBP-GFP-TgRab11A was lowly expressed and its localization was not disturbed. In this condition, the parasites grew normally. TgRab11A partially colocalized with TgStx20 and TgStx21 at the apical annuli (Fig 4A). When TgStx20 or TgStx21 was depleted, TgRab11A signals significantly aggregated at the apical pole of the parasites (Fig 4B and 4C). Further co-localization analysis indicated that the sites where TgRab11A accumulated were identical to the apical annuli (Fig 4D). To exclude the possibility that TgRab11A accumulation was caused by an artificial effect of overexpression, we inserted a GFP tag at the N-terminus of endogenous TgRab11A using the CRISPR/Cas9 method in the strain expressing AID*-tagged TgStx21. GFP-TgRab11A signals also accumulated at the apical annuli when TgStx21 was depleted (S4 Fig). These phenomena indicate that trafficking of vesicles decorated with TgRab11A was congested at the apical annuli when TgStx20 or TgStx21 was degraded. If this was the case, after removing auxin, the accumulated signals of TgRab11A would disappear when vesicular fusion mediated by TgStx20 or TgStx21 recovered. We then performed live cell microscopy to record the fate of the aggregated puncta when IAA was washed out from the culture medium. The accumulated signals of TgRab11A caused by depletion of TgStx21 gradually decreased after IAA was washed away (Fig 4E, S1 and S2 Movies). These results strongly indicate that certain vesicular fusion events mediated by TgStx20 and TgStx21 occur at the apical annuli and are associated with TgRab11A.

### TgStx1 forms a complex with TgStx20 and TgStx21 and is required for the lytic cycle of *T*. *gondii*

**Fig 5:**
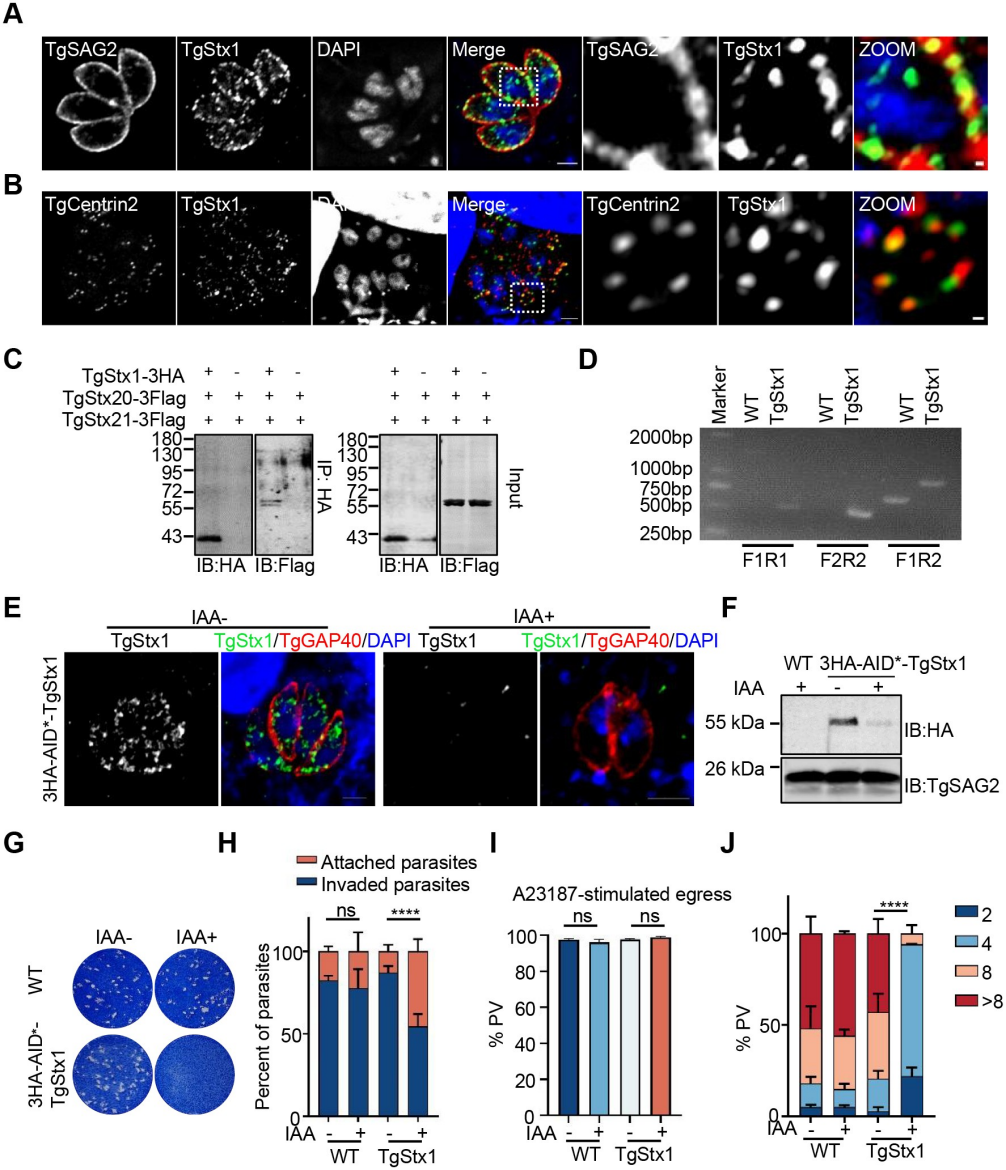
TgStx1 is essential for the lytic cycle of *T*. *gondii*. **(A)** IFA showing that TgStx1 (green) partially colocalized with the plasma membrane protein TgSAG2 (red). Scale bars: 2 μm (left panels) and 0.2 μm (zoomed panels). **(B)** IFA showing that 3HA-AID*-TgStx1 (red) partially colocalized with Tgcentrin2 (green). 3HA-AID*-TgStx1 parasites were transiently transfected with a plasmid expressing TgCentrin2 fused with EGFP and subjected to an IFA. Scale bars: 2 μm (left panels) and 0.2 μm (zoomed panels). (**C**) Co-immunoprecipitation of 3HA-tagged TgStx1 and 3Flag-tagged TgStx20 and TgStx21. HEK293T cells transiently expressing 3HA-TgStx1, 3Flag-TgStx20, and 3Flag-TgStx21 were subjected to immunoprecipitation. IP, immunoprecipitation; IB, immunoblot. (**D**) Genomic PCR analysis confirming homologous recombination of the genomic 3HA-AID*-TgStx1 locus using the indicated primers. (**E**) 3HA-AID*-TgStx1 parasites were transiently transfected with a plasmid expressing TgGAP40-YFP, cultured with or without IAA for 24 h, and stained with an antibody against HA (green). Scale bars: 2 μm. (**F**) The parasites were cultured in the presence or absence of IAA for 12 h *in vivo* and lysed. The expression of AID*-tagged TgStx1 was evaluated by WB analysis. (**G**) Plaque assays were performed by infecting BJ-5ta cells with 3HA-AID*-TgStx1 parasites for 6 d in the presence or absence of IAA. (**H**) Invasion assay examining the ability of 3HA-AID*-TgStx1 parasites to attach to and invade host cells in the presence or absence of IAA. Parasites were grown for 6 h in the presence or absence of IAA before the invasion. The data were analyzed for statistical significance by the unpaired t-test; ****P<0.0001. (**I**) Egress of 3HA-AID*-TgStx1 parasites from host cells was determined by an IFA. Parasites were grown in BJ-5ta cells for 36 h and treated with 500 nM IAA or vehicle (EtOH) for 8 h prior to stimulation with A23187 for 5 min. Statistical differences were analyzed by the unpaired t-test; ns. (**J**) Replication of 3HA-AID*-TgStx1 parasites after growth in the presence or absence of IAA for 24 h. The average number of parasites per vacuole was determined. The data were analyzed by a two-way ANOVA, ****P<0.0001.

SNAP23 functions together with Stx1 and VAMP8 at the plasma membrane in exocytotic membrane fusion. Two SNAREs, TgStx1 (TGGT1_209820) and TgVAMP8 (TGGT1_257520), were found in the plasma membrane of *T*. *gondii* in our previous study [18]. An IFA revealed that TgStx1 partially localized with TgSAG2 at the plasma membrane, and similar to TgStx20 and TgStx21, colocalized with TgCentrin2 at the apical annuli (Fig 5A and 5B). The interactions of TgStx1 with TgStx20 and TgStx21 were confirmed by immunoprecipitation assays using recombinant proteins expressed in HEK293 cells (Fig 5C). These results indicate that TgStx1 forms a complex with TgStx20 and TgStx21. The AID conditional knockout system was also used to create a TgStx1-knockout strain. To confirm the correct integration, diagnostic PCR analyses and sequencing were conducted. Only three HAs fused with an AID* tag could be integrated at the N-terminus of TgStx1 (Fig 5D). An IFA and WB found that 3HA-AID*-tagged TgStx1 was degraded in the presence of IAA (Fig 5E and 5F). To explore the effect of gene deletion on the growth of parasites, we first performed the plaque assays described above. The TgStx1 strain did not form plaques in HFFs after 8 days of incubation with IAA, but formed normal plaques in the absence of IAA (Fig 5G). These data indicate that depletion of TgStx1 severely affects the growth of these parasites. Invasion of host cells by the parasites was also affected when the expression of TgStx1 was abolished, but the ability to egress was not (Fig 5H and 5I). The replication assays revealed that PVs rarely contained four parasites (Fig 5J). These data indicate that depletion of TgStx1 severely affects the lytic cycle of these parasites.

### TgStx1 is needed for protein exocytosis at the plasma membrane and Rab11A-positive vesicle fusion at the apical annuli

**Fig 6:**
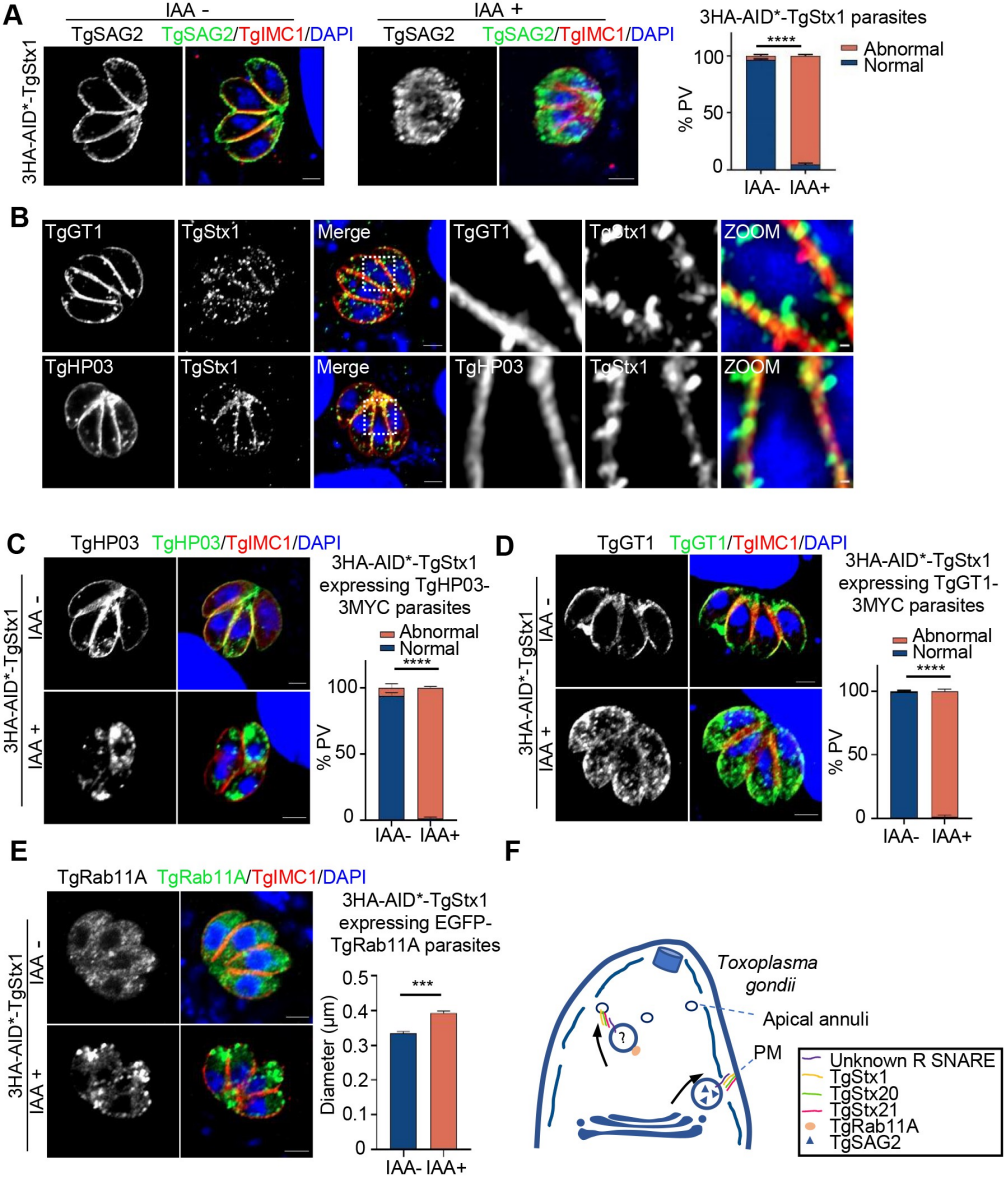
TgStx1 is required for exocytosis at the plasma membrane. (**A**) IFA showing that conditional ablation of TgStx1 affected the trafficking of TgSAG2 (green) to the plasma membrane. The parasite cortex was stained with a rabbit anti-TgIMC1 antibody (red). Scale bars: 2 μm. Data show mean ± SEM of three independent experiments and statistical differences were analyzed by the unpaired t-test; ****P<0.0001. (**B**) IFA showing that TgStx1 (green) partially colocalized with TgHP03 (red) and TgGT1 (red). 3HA-AID*-TgStx1 parasites were transfected with a plasmid expressing 3MYC-tagged TgHP03 or TgGT1, selected using pyrimethamine, and then analyzed by an IFA. Scale bars: 2 μm (left panels) and 0.2 μm (zoomed panels). (**C**) and (**D**) IFA showing that TgHP03 (green) and TgGT1 (green) were retained in intra-cytosolic vesicles following treatment of 3HA-AID*-TgStx1 parasites with IAA for 24 h, but were located at the membrane without IAA treatment. The parasite cortex was stained with a rabbit anti-TgIMC1 antibody (red). Scale bars: 2 μm. Data show mean ± SEM of three independent experiments and statistical differences were analyzed by the unpaired t-test; ****P<0.0001. (**E**) IFA showing that TgRab11A (green) aggregated at the apical annuli following treatment of 3HA-AID*-TgStx1 parasites with IAA for 24 h, but was distributed in the cytoplasm without IAA treatment. The parasites were transfected with a plasmid expressing FKBP-EGFP-TgRab11A, selected using pyrimethamine, and then analyzed by an IFA. The parasite cortex was stained with a rabbit anti-TgIMC1 antibody (red). The longest diameters of the TgRab11A puncta were measured using the distance tool of ZEN 3.3 software. One hundred Rab11A-positive puncta at the apical pole were measured in each slide. Data shown in the graphs are the mean ± SEM of three independent experiments and statistical differences were analyzed by the unpaired t-test; ***P = 0.0002. Scale bars: 2 μm. (**F**) A proposed model to explain how TgStx1, TgStx20, and TgStx21 participate in vesicular fusion at the plasma membrane and apical annuli.

We then examined exocytosis of the surface protein TgSAG2 in 3HA-AID*-TgStx1 parasites. TgSAG2 presented as puncta and was diffuse in the cytoplasm when TgStx1 was absent (Fig 6A). Next, we examined the role of TgStx1 in the trafficking of TgHP03 and TgGT1. TgStx1 partially colocalized with the transmembrane proteins TgHP03 and TgGT1 at the plasma membrane (Fig 6B). A lack of TgStx1 affected the localization of TgGT1 and TgHP03 on the plasma membrane (Fig 6C and 6D). The role of TgStx1 at the apical annuli was investigated by overexpressing TgRab11A in the 3HA-AID*-TgStx1 strain. Depletion of TgStx1 caused significant aggregation of TgRab11A at the apical annuli (Fig 6E). The secretion of TgGRA7 was also investigated in TgStx1-deficient parasites. Similar to the observations in TgStx20- or TgStx21-depleted parasites, exocytosis was unaffected (S5 Fig).

These data indicate that TgStx1, TgStx20, and TgStx21 form a critical SNARE complex that governs the exocytotic process of surface molecules and transmembrane proteins in *T*. *gondii*. Moreover, these SNAREs are also required for TgRab11A-associated vesicular fusion at the apical annuli (Fig 6F).

## Discussion

The molecular mechanism underlying exocytosis in *T*. *gondii* remained unclear until now, although it is an essential process for successful invasion and replication in host cells. In this study, we revealed that an unconventional SNARE complex mediates exocytosis at the plasma membrane in *T*. *gondii*.

*T*. *gondii* membranes not only contain multiple transporters responsible for material exchange to facilitate the movement of small solutes, such as metabolites, oligosaccharides, and amino acids, across cell membranes according to chemiosmotic gradients [27], but also serve as a platform to mediate the fusion of secreted organelle contents with the plasma membrane. However, little is known about the exocytotic step of these proteins. SNAP25 family proteins (SNAP23 and SNAP25) function in the fusion of secretory vesicles with the targeting membrane in other organisms [28,29]. The Qb and Qc SNARE motifs of conventional SNAP25 are connected by a linker region containing five palmitoylated cysteines that anchor it at the plasma membrane [30]. Integration of two SNARE motifs in a single molecule of SNAP25 family proteins may increase the speed and precision of binding to another molecule and thereby facilitate fusion. In *T*. *gondii*, however, only one palmitoylation site-free SNAP25 family protein (TgSNAP29), which is responsible for the transport of apicoplast proteins, has been identified [14]. Instead, two SNAREs (TgStx20 and TgStx21) share homology with the C-terminal Qc and N-terminal Qb SNARE domains of SNAP23. In this study, we found that TgStx20 and TgStx21 interact with TgStx1 at the plasma membrane and apical annuli and are required for the proper localization of surface and transmembrane proteins at the plasma membrane.

Successful infection of *T*. *gondii* relies on sequential exocytosis of secreted organelles, including micronemes, rhoptries, and dense granules. However, no SNARE proteins that localize to micronemes and rhoptries have been identified in *T*. *gondii*. It is suggested that C2 domain-containing proteins (TgDOC2 and TgFER2) may mediate microneme exocytosis at the plasma membrane without SNAREs [31,32]. In the case of the secretion of dense granules, it is reported that exocytosis of GRAs may occur at the apical pole and be mediated by TgRab11A [15]. In our experiments, none of the SNAREs were shown to be related to the exocytosis of GRAs. Therefore, the mechanism that mediates exocytosis of GRAs at the plasma membrane remains to be elucidated. However, it is possible that the exocytic mechanism of GRAs in intracellular parasites might be different. In particular, a burst of GRA exocytosis has been observed shortly after the completion of host cell invasion [33]. We cannot exclude the possibility that these SNAREs might be required for a burst in secretion of GRAs. In addition, the extent of SNARE depletion using the AID system might be insufficient to block the secretion of GRAs. Another explanation is that various SNAREs might participate in the exocytotic process required for the secretion of GRAs. Depletion of one of these SNAREs will not cause a severe deficiency of exocytosis. Exocytotic deficiency is not always observed in other organisms when exocytosis-associated SNAREs are depleted [34].

The function of the apical annuli is unclear in *T*. *gondii*. The annuli are approximately 200 nm in diameter, and normally 5–6 annuli are observed in one parasite. This structure is composed of coiled-coil and signaling proteins, which are proposed to assemble into pore-like structures in the IMC and are believed to play a role in replication [35]. The finding that a SNARE complex comprising TgStx1, TgStx20, and TgStx21 exists at the apical annuli indicates that vesicular fusion occurs at these sites. To the best of our knowledge, TgStx20 and TgStx21 are the first transmembrane proteins discovered at these structures, which suggests that the apical annuli are parts of the membrane or form closed compartments rather than pore-like structures. Moreover, the annuli structures are embedded in the IMC, and there is currently no clear evidence of a connection between the apical annuli and the plasma membrane. Therefore, the vesicular fusion events mediated by TgStx20 and TgStx21 at the apical annuli are likely unrelated to exocytosis. Furthermore, the vesicular fusion events at the apical annuli are not necessarily related to the secretion of surface and transmembrane proteins. Most likely, the vesicular fusion events mediated by the TgStx20-TgStx21 complex are required to properly position apical annuli-residing proteins at these sites or deliver cargoes that cross these structures.

Our data indicate that depletion of TgStx20 or TgStx21 leads to accumulation of TgRab11A signals around the apical annuli, but not in the plasma membrane. This result suggests that the docking process at the plasma membrane might be different from that at the apical annuli, which could explain why Rab11A-positive vesicles accumulated only at the apical annuli when TgStx20 or TgStx21 was depleted. Another possible explanation is that vesicular fusion is much more active at the apical annuli than at the plasma membrane. Consequently, the dissociation of TgRab11A could not be executed rapidly enough at the apical annuli. The association of TgRab11A with the secretion of surface and transmembrane proteins has been unambiguously demonstrated. The accumulated signals of TgSAG2 and TgGT1 in the cytoplasm were not decorated with TgRab11A, which indicates that TgRab11A was disassociated from the secretory vesicles by guanosine dissociation inhibitors.

Like other protozoa, the number of Qa SNAREs is reduced in *T*. *gondii*. TgStx1 was the only Qa SNARE that clearly localized to the plasma membrane in our experimental conditions. As expected, TgStx1 was involved in the secretion of GPI-anchored surface antigens and the trafficking of transporters to the membrane. Our previous study reported that the SM protein TgSec1c [18], whose ortholog is a vesicular pathway mediator and serves as a platform to assist the assembly of SNARE complexes [36], is also involved in the secretion of GPI-anchored surface antigens. It is necessary to investigate whether the assembly of the TgStx1-TgStx20-TgStx21 complex depends on TgSec1c. Moreover, SNAP23 anchors to membranes through palmitoylation sites. By contrast, TgStx20 and TgStx21 contain transmembrane domains, which suggests that the anchoring mechanism of these two SNAREs at the plasma membrane and apical annuli might be different.

In conclusion, this study revealed unconventional SNAREs involved in exocytosis in *T*. *gondii*. These SNAREs mediate vesicular fusion together with the conserved Qa SNARE TgStx1. This unique SNARE complex functions in vesicular fusion at the plasma membrane and apical annuli of *T*. *gondii*.

## Materials and methods

### Gene identification and phylogeny

The potential domains in the sequences of *T*. *gondii* (TgStx20 [TGGT1_253360], TgStx21 [TGGT1_306640], and TgSNAP29 [TGGT1_319940]), *Saccharomyces cerevisiae* (Sec9p [YGR009C] and Spo20p [YMR017W]), and human (SNAP23 [HGNC:11131], SNAP25 [HGNC:11132], and SNAP29 [HGNC:11133]) proteins were analyzed by multiple sequence alignment using CLUSTALW (https://www.genome.jp/tools-bin/clustalw). All phylogenetic trees were constructed by the PhyML algorithm with the default parameters and rooted at the midpoint. Branch supports were computed from 100 bootstrapped trees.

### Parasite strains and culturing

The TIR1-expressing RHΔKU80 strain [37,38] was grown on Vero cells or the hTERT-immortalized HFF cell line BJ-5ta (ATCC CRL-4001), which were cultured in complete Dulbecco’s Modified Eagle’s Medium (DMEM, Sigma-Aldrich, D6429) supplemented with 10% (v/v) fetal bovine serum (CLARK, FB25015), 20% (v/v) Medium 199 Earle’s Salts (ThermoFisher Scientific, C11150500BT), and 1% penicillin/streptavidin.

### IFA

Confluent HFF monolayers were cultured on φ15 mm tissue culture dishes (Nest, 801007) or coverslips and infected with parasites prior to fixation with formaldehyde prepared in phosphate-buffered saline (PBS, Biosharp, BL302A) and permeabilization with 0.3% Triton X-100 (Solarbio Life Sciences, T8200) prepared in PBS for 10 min. The dishes were then blocked with 5% bovine serum albumin (Solarbio Life Sciences, T8020) prepared in PBS and incubated overnight with primary antibodies diluted with blocking buffer. After five washes with Tris-buffered saline (20 mM Tris-HCl pH 8.0 and 150 mM NaCl [Amresco, 0497; Sinopharm Chemical Reagent Co., Ltd, 10019328]) containing 0.5% Tween-20 (Amresco, 0777), the dishes were stained with the appropriate secondary antibodies and DAPI (Sigma-Aldrich, D9542). The samples were observed on an LSM880 confocal microscope (Zeiss) with a 63×/1.4 oil objective or an LSM980 microscope (Zeiss) with a 100×/1.4 oil objective. Images were acquired using a Zeiss Zen Blue 2.3 Lite system equipped with an Airyscan module.

### Plasmid construction

PCR was performed using KOD-Plus-Neo (CFKOD401, Toyobo) or TaKaRa Ex Taq (RR001, Takara). The SmFP-HA and SmFP-MYC tags were synthesized by GenScript (Jiangsu). The AID* sequence was amplified from pLinker-AID-3xHA-HXGPRT-LoxP (Addgene #86553). The guide RNA sequences specific for TgStx1 (aatttccacgcatgcatgcg), TgStx20 (aacgaagatgaacgtgacca), TgStx21 (ggtcacgtccaagatgagtg), TgAAP4 (aacagtgaagaagagtgccg), and TgRab11A (ttagccgccatggtgaagag) were designed by EuPaGDT (http://grna.ctegd.uga.edu/) and cloned into the PmeI site of pCD-Cas9 [37] to construct CRISPR/Cas9 plasmids. The open reading frames of TgGT1-3MYC, TgHP03-3MYC, TgSORTLR-EGFP, TgISP1-EGFP, TgMIC6-3MYC, TgGAP40-YFP, and FKBP-GFP-TgRab11A were amplified and introduced into the pBS-DHFR vector under the control of the Tgβtubulin or TgGRA1 promoter. To express TgStx1, TgStx20, and TgStx21 in HEK293T cells, the open reading frames were cloned into the pCAGGS vector with 3×HA or 3×FLAG, as indicated in the figures.

### Reagents and antibodies

IAA (Aladdin, I101074) was prepared at a concentration of 500 mM in absolute ethanol. Mouse and rabbit anti-TgSAG2 (1:2000), rabbit anti-TgTubulin (1:2000), rabbit anti-TgIMC1 (1:2000), mouse anti-TgGRA7 (1:500), and rabbit anti-TgMIC2 (1:500) antibodies used for the IFA and WB were prepared in our laboratory (39). Commercial mouse anti-HA monoclonal (Sigma-Aldrich, H9658), mouse anti-FLAG monoclonal (Sigma-Aldrich, F3165), rabbit anti-HA monoclonal (Cell Signaling Technology, 3724S), rabbit anti-FLAG monoclonal (Cell Signaling Technology, 14793S), mouse anti-c-MYC (Cell Signaling Technology, 2276S), and rabbit anti-c-MYC monoclonal (Cell Signaling Technology, 2278S) antibodies were diluted 1:1000 for the IFA and 1:2000 for WB. Alexa Fluor 488-coupled (Invitrogen, A11001/A11034) and Alexa Fluor 594-coupled (Invitrogen, A11037/A11032) secondary antibodies were diluted 1:1000 for the IFA. DyLight 800-labeled anti-mouse IgG (SeraCare Life Sciences, 5230-0415/ 5230–0412) was diluted 1:10,000 for WB.

### Transfection and selection of parasites

To obtain transgenic parasites, parasites (10^7^) were placed in a 2 mm gap cuvette (Bio-Rad, 1652089), after which electroporation was performed using standard procedures at 1.5 kV, 50 Ω, and 25 μF with a BTX electroporator. Transgenic parasites were allowed to grow in Vero cells for 24 h and then sorted by flow cytometry. Sorted cells were inoculated into 96-well plates with fibroblast monolayers. On day 7 after sorting, clones were screened by PCR to confirm the correct integration.

### Construction of conditional TgStx1-, TgStx20-, and TgStx21-knockout parasites

To construct AID inducible conditional knockout strains, the TIR1-3FlagΔKU80 strain was co-transfected with a linearized DNA fragment and the pCD-Cas9 plasmid to tag the endogenous genomic locus of TgStx1, TgStx20, or TgStx21 with 12HA-AID* at the N-terminus. DNA for electroporation (≥ 5 μg of PCR product flanked by 40 bp homology arms of the translation start/stop codon) was amplified by KOD polymerase along with up to 30 μg of the CRISPR/Cas9 plasmid.

### Plaque assays

Monolayers of HFFs grown in 12-well plates were inoculated with 200 tachyzoites per well and then cultured with or without IAA. After 36 h, the culture medium was replaced with fresh medium, and the cells were cultured continuously for another 7 d. The culture was then fixed with cold methanol and stained with Coomassie Brilliant Blue G250 (Solarbio, C8430-5).

### Invasion assay

Parasites were pretreated with or without IAA for 12 h and added to monolayers of Vero cells grown in 12-well plates and allowed to invade for 3 h under normal growth conditions. After washing the monolayers three times with PBS to remove extracellular parasites, cells were fixed with 4% formaldehyde (Biosharp, BL539A). Adhered parasites were labeled with a rabbit anti-TgSAG2 antibody and visualized with Alexa Fluor 488-coupled anti-rabbit IgG without permeabilization. Cells were permeabilized by incubation with 0.3% Triton X-100 prepared in PBS for 15 min at room temperature. Intracellular and extracellular parasites were labeled with a rabbit anti-TgSAG2 antibody and visualized with Alexa Fluor 594-coupled anti-rabbit IgG. One hundred parasites were counted in each slide and the number of invaded parasites (green) was calculated by subtracting the number of adhered parasites (co-labeled) from the total number of parasites. Results are from three independent biological experiments.

### Intracellular replication assay

Freshly egressed parasites were added to monolayers of HFFs in 12-well plates and allowed to grow with or without IAA for 24 h. Following three washes with PBS to remove extracellular parasites, cells were fixed with 4% paraformaldehyde and parasites were detected by an IFA using an anti-TgSAG2 antibody and visualized with Alexa Fluor 488-coupled anti-rabbit IgG. The number of parasites per vacuole was determined, with 100 vacuoles examined per condition. Results are from three independent biological replicates.

### Induced egress assay

Freshly egressed parasites were added to monolayers of HFFs in 12-well plates and allowed to grow for 20 h. Parasites were then cultured in media containing or lacking IAA for 16 h before 3 μM Ca^2+^ ionophore A23187 (Sigma-Aldrich, C9275-1MG-QM) was added to induce egress. After incubation in a water bath at 37°C for 5 min, parasites were fixed and an IFA was performed with rabbit anti-SAG2 and mouse anti-GRA7 antibodies. A total of 100 vacuoles were counted per field.

### WB and immunoprecipitation

For immunoblotting, parasites and cells were collected, lysed with RIPA buffer (Sigma-Aldrich, R0278) or lysis buffer (20 mM Tris-HCl pH 8.0, 2 mM EDTA, 150 mM NaCl, and 1% Triton X-100) for 30 min on ice, and then centrifuged at 13,000 g for 30 min. The lysate was loaded onto a 10% SDS polyacrylamide gel. Proteins were blotted onto a PVDF membrane and probed with the indicated primary antibodies followed by species-specific secondary antibodies. Antibodies were diluted in 5% milk dissolved in Tris-buffered saline containing 0.5% Tween-20. The probed PVDF membranes were visualized using Odyssey CLX (Li-Cor).

For immunoprecipitation, HEK293T cells at 70–80% confluency were transfected with plasmids harboring TgStx1-3HA, TgStx20-3FLAG, and TgStx21-3FLAG, collected after 48 h, and lysed in lysis buffer. The supernatant (100 μl) was used for input analysis. The remaining supernatant (400 μl) was mixed and incubated with EZview Red Anti-HA Affinity Gel (Sigma-Aldrich, E6779) or Anti-FLAG M2 Magnetic Beads (Sigma-Aldrich, M8823) for 1 h or overnight at 4°C with gentle rotation. The beads were washed five times with lysis buffer, and bound proteins were eluted with SDS-PAGE sample buffer.

### Secretion assay

Parasite cultures were preincubated for at least 12 h with or without IAA before egression and then 1×10^8^ extracellular tachyzoites were pelleted by centrifugation at 1000 × g for 5 min at room temperature. Samples were washed with pre-warmed serum-free DMEM, resuspended in 100 μl of serum-free DMEM (GRAs), and then immediately incubated in a water bath at 37°C for 30, 60, and 90 min. Following pelleting by centrifugation at 1000 × g for 5 min at 4°C, the supernatant was transferred to a new tube (the pellet from this step served as the pellet fraction) and re-pelleted at 2000 × g for 5 min at 4°C. The final supernatant, containing excreted secreted antigens (ESAs), and pellet fractions were lysed in an equal volume of RIPA buffer for 30 min on ice and separated by SDS-PAGE.

### Metabolomic analysis

To incorporate ^13^C into metabolites of host cells, a monolayer of HFFs cultured in a normal medium was washed with PBS and then cultivated in glucose-free DMEM (BBI, E600010-0500) supplemented with 8 mM 13C6-glucose (SIGMA, 389374) for 3 days. Parasites were then added to a monolayer of HFFs grown for 36 h with or without IAA, forcibly released and purified from host cells/debris by passage through a 26 gauge needle, filtered through a 5 μm polycarbonate filter to remove host debris, and pelleted by centrifugation at 1000 ×g for 10 min. A total of 10 mg of precipitate was analyzed by a gas chromatography-mass spectrometry-based non-targeted metabolomics platform. The cell samples were supplemented with a 60-fold volume (vol/wt, μL/mg) of cold 80% aqueous methanol (1,4, v/v) and subjected to five cycles comprising ultrasonication for 2 min and an interval of 2 min in an ice-water bath. After centrifugation at 15,000 rcf and 4°C for 15 min, 400 μL of the supernatant and 10 μL of L-norleucine (50 μg/mL) were mixed and evaporated to dryness under a nitrogen stream. The residue was reconstituted in 30 μL of 20 mg/mL methoxyamine hydrochloride in pyridine, and the resulting mixture was incubated at 37°C for 90 min. Thereafter, 30 μL of N, O-bis (trimethylsilyl)trifluoroacetamide (BSTFA) (with 1% TMCS) was added to the mixture and derivatized at 70°C for 60 min prior to gas chromatography-mass spectrometry-based metabolomics analysis.

For multivariate statistical analysis, the normalized data were imported into SIMCA software (version 14.1; AB Umetrics, Umeå, Sweden). The data were preprocessed by unit-variance scaling and mean centering before performing principal component analysis, partial least squares-discriminant analysis, and orthogonal partial least squares-discriminant analysis (OPLS-DA). The variables with VIP values in the OPLS-DA model higher than 1 and p values in univariate statistical analysis lower than 0.05 were identified as potential differential metabolites. The heatmap analysis of the differential metabolites was conducted through an R platform (Version 3.4.3). The pathway analysis was performed using the online software MetaboAnalyst (version 4.0, http://www.metaboanalyst.ca/).

BSTFA (with 1% TMCS), methoxyamine hydrochloride, methanol, and anhydrous pyridine were purchased from Sigma-Aldrich. For gas chromatography-mass spectrometry, Agilent 7890A gas chromatography and Agilent 5975C inert MSD systems were used. The chromatographic column used was an OPTIMA 5 MS Accent fused-silica capillary column (30 m × 0.25 mm × 0.25 μm; MACHEREY-NAGEL, Düren, Germany). Internal standards, including L-norleucine, were purchased from Sigma-Aldrich.

Sample processing and analysis were completed by Shanghai Spectrum Biological Technology Co., Ltd.

### Oligonucleotides

All primers used in this study are listed in S1 Table.

### Statistical analyses

Data were analyzed using GraphPad Prism 9 software. Data are presented as the mean ± SD from triplicate, parallel, independent experiments unless otherwise indicated. All statistical analyses were performed using the Student’s t-test or a two-way ANOVA unless otherwise specified.

## Supporting information

S1 Fig Immunofluorescence analysis of proteins residing at the ER, Golgi, Micronemes, and IMC in TgStx21-depleted parasites. 12HA-AID*-TgStx21 parasites were transiently transfected with plasmids expressing TgSAG1-GPI-HDEL-EGFP (**A**), TgSORTLR-EGFP (**B**), TgMIC6-3MYC (**C**), or TgISP1-EGFP (**D**) under the control of the Tgβtubulin promoter and cultured in the presence or absence of IAA for 24 h. The marker proteins were stained with anti-MYC and anti-EGFP antibodies (green) and the parasite cortex was stained with an anti-TgIMC1 antibody (red). Scale bars: 2 μm.
(PDF)

S2 Fig Metabolic pathways affected by depletion of TgStx21. (**A**) Heatmap analysis of quantitative information about differential metabolism in the presence and absence of IAA. Each row indicates a differential metabolite, each column indicates the sample number, and the tree structure on the left indicates the similarity clustering relationship between the differential metabolites. Red and blue indicate that the concentration of the differential metabolite in the sample is increased and decreased, respectively. (**B**) The extent of the metabolic pathway’s influence is shown by the size of the circle in the illustration.
(PDF)

S3 Fig Exocytosis of GRA proteins is unaffected by loss of TgStx21. (**A**) TgGRA7 secretion in 12HA-AID*-TgStx21 parasites treated with or without IAA for 12 h was examined by WB. TgTubulin was used as a loading control. (**B**) Secreted TgGRA7 (ESA fraction) and intracellular TgGRA7 (pellet fraction) were quantified from three independent experiments. (**C**) Immunofluorescence staining of TgGRA7 (green), TgGRA2 (green), GRA52 (green), TgGRA24 (green), and TgIMC1 (red) in 12HA-AID*-TgStx21 parasites treated with or without IAA for 24 h. 12HA-AID*-TgStx21 parasites were transfected with plasmids expressing 7MYC-tagged TgGRA2 or 3MYC-tagged GRA52 and selected using pyrimethamine. Scale bars: 2 μm. (**D**) Co-localization analysis of GRA52 (green) and TgGRA7 (red) in extracellular parasites. Scale bars: 2 μm.
(EPS)

S4 Fig TgRab11A signals accumulate at the apical pole in TgStx21-depleted parasites. An EGFP tag was inserted at the N-terminus of endogenous TgRab11A using the CRISPR/Cas9 method. EGFP signals (green) were detected in 12HA-AID*-TgStx21 parasites treated with or without IAA for 24 h. The parasite cortex was stained with a rabbit anti-TgIMC1 antibody (red). Scale bars: 2 μm.
(PDF)

S5 Fig Exocytosis of TgGRA7 is unaffected by loss of TgStx1. (**A**) TgGRA7 secretion in 3HA-AID*-TgStx1 parasites treated with or without IAA for 24 h was examined by WB. TgTubulin was used as a loading control. (**B**) Secreted TgGRA7 (ESA fraction) and intracellular TgGRA7 (pellet fraction) were quantified from three independent experiments. (**C**) Immunofluorescence staining of TgGRA7 (green), TgGRA2 (green), GRA52 (green), and TgIMC1 (red) in 3HA-AID*-TgStx1 parasites treated with or without IAA for 24 h. 3HA-AID*-TgStx1 parasites were transfected with plasmids expressing 7MYC-tagged TgGRA2 or 3MYC-tagged GRA52 and selected using pyrimethamine. Scale bars: 2 μm.
(EPS)

S1 Table Primers used in this study. (DOCX)

S1 Movie Dynamics of TgRab11A-positive vesicles (green) in intracellular 12HA-AID*-TgStx21 parasites stably expressing FKBP-EGFP-TgRab11A in the absence of IAA. The parasites were cultured in the presence of IAA for 24 h. The IAA was then washed out and cultured for 1 h before imaging. Imaging speed: 0. 2 fps.
(AVI)

S2 Movie Dynamics of TgRab11A-positive vesicles (green) in intracellular 12HA-AID*-TgStx21 parasites stably expressing FKBP-EGFP-TgRab11A in the presence of IAA. The parasites were cultured in the presence of IAA for 24 h and used for imaging directly. Imaging speed: 0. 2 fps.
(AVI)
