# Supplementary figures and images for: An unconventional SNARE complex mediates exocytosis at the plasma membrane and vesicular fusion at the apical annuli in Toxoplasma gondii
Source: PLoS Pathog. 2023 Mar 27;19(3):e1011288. doi: 10.1371/journal.ppat.1011288 (PMC10079086; doi:10.1371/journal.ppat.1011288)

Figure S1 Fu et al.

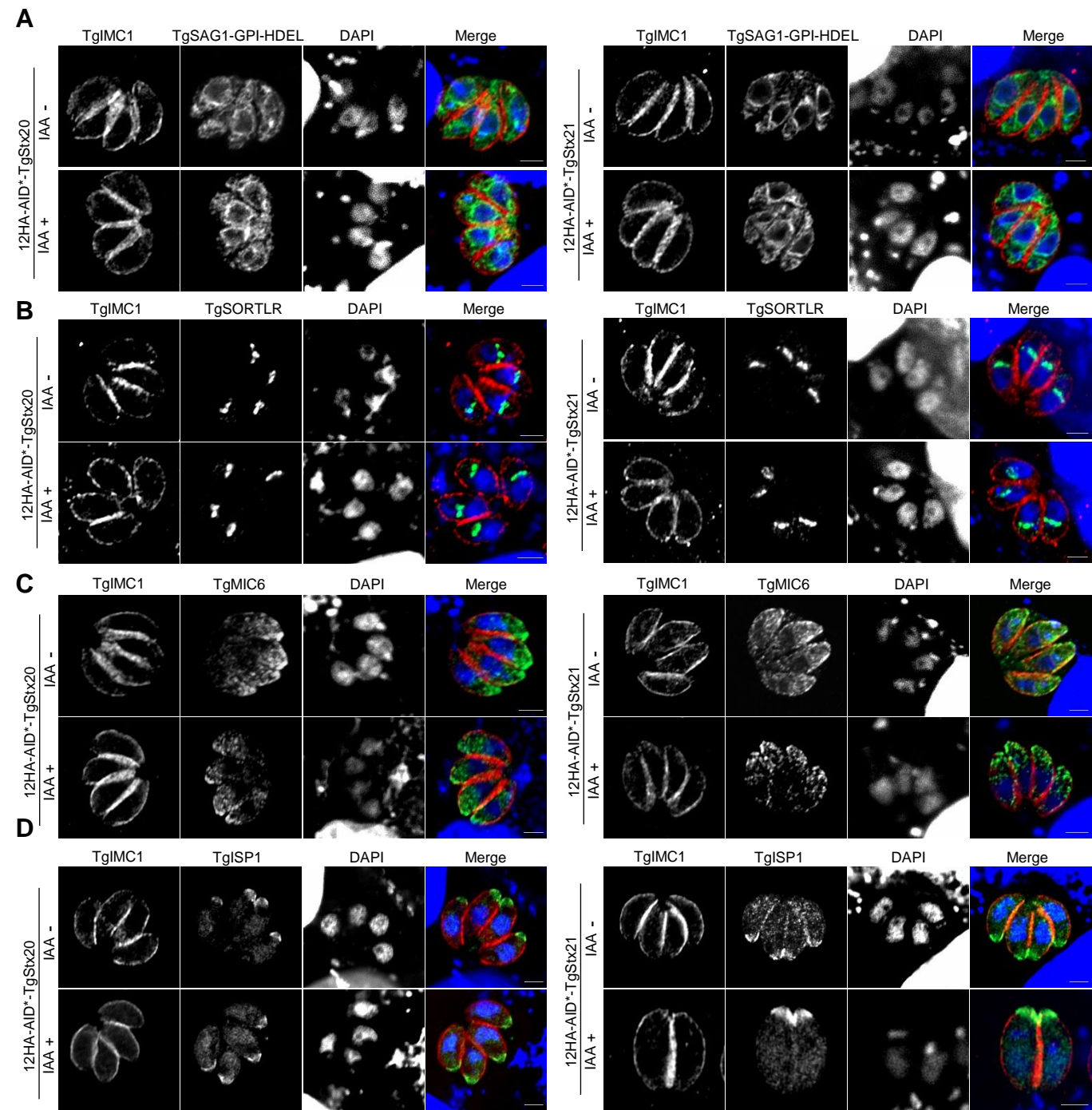

Supplement: S1 Fig — 12HA-AID*-TgStx21 parasites were transiently transfected with plasmids expressing TgSAG1-GPI-HDEL-EGFP (A), TgSORTLR-EGFP (B), TgMIC6-3MYC (C), or TgISP1-EGFP (D) under the control of the Tgβtubulin promoter and cultured in the presence or absence of IAA for 24 h. The marker proteins were stained with anti-MYC and anti-EGFP antibodies (green) and the parasite cortex was stained with an anti-TgIMC1 antibody (red). Scale bars: 2 μm. (PDF) [file ppat.1011288.s001.pdf]

Figure S2 Fu et al.

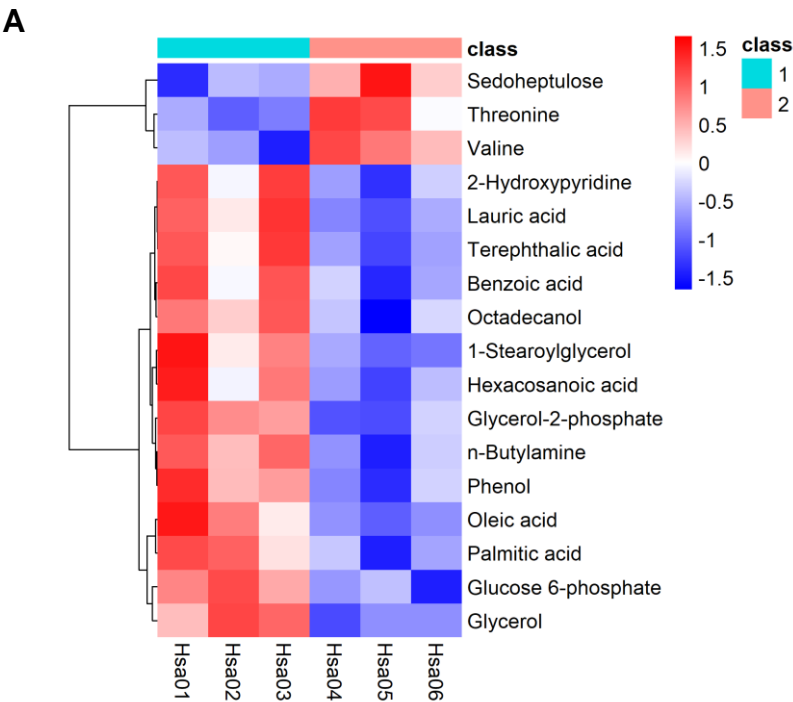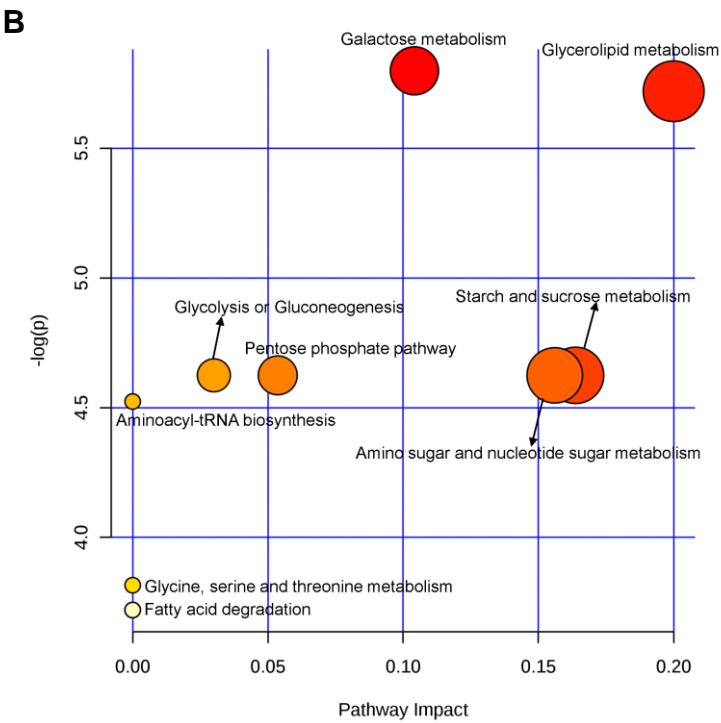

Supplement: S2 Fig — (A) Heatmap analysis of quantitative information about differential metabolism in the presence and absence of IAA. Each row indicates a differential metabolite, each column indicates the sample number, and the tree structure on the left indicates the similarity clustering relationship between the differential metabolites. Red and blue indicate that the concentration of the differential metabolite in the sample is increased and decreased, respectively. (B) The extent of the metabolic pathway’s influence is shown by the size of the circle in the illustration. (PDF) [file ppat.1011288.s002.pdf]

Figure S4 Fu et al.

A

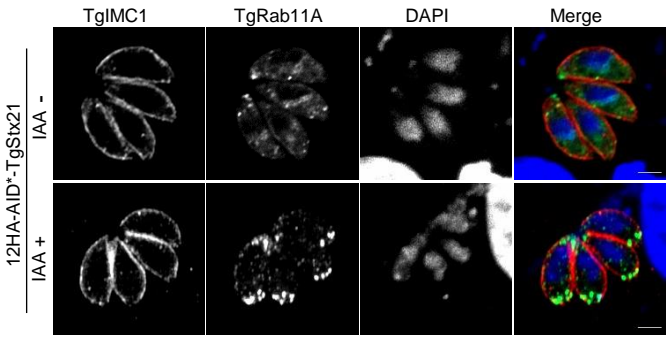

Supplement: S4 Fig — An EGFP tag was inserted at the N-terminus of endogenous TgRab11A using the CRISPR/Cas9 method. EGFP signals (green) were detected in 12HA-AID*-TgStx21 parasites treated with or without IAA for 24 h. The parasite cortex was stained with a rabbit anti-TgIMC1 antibody (red). Scale bars: 2 μm. (PDF) [file ppat.1011288.s004.pdf]
