# Supplementary material for: An unconventional SNARE complex mediates exocytosis at the plasma membrane and vesicular fusion at the apical annuli in Toxoplasma gondii
Source: PLoS Pathog. 2023 Mar 27;19(3):e1011288. doi: 10.1371/journal.ppat.1011288 (PMC10079086; doi:10.1371/journal.ppat.1011288)
Supplement: S1 Table — (DOCX) [file ppat.1011288.s006.docx]

**Supplementary data**

**Table S1.** Primers used in this study.

| **Primers** | **Sequences（5‘-3’）** | **Purposes** |
| --- | --- | --- |
| Cas9-F | ACTAGTTCTAGAGCGGGA | Cas9 plasmid |
| gRNA-TgStx1-R | CTATTTCTAGCTCTAAAACCGCATGCATGCGTGGAAATTAACTTGACATCCCCATTTAC | TgStx1 Cas9 plasmid |
| gRNA-TgStx20-R | CTATTTCTAGCTCTAAAACTGGTCACGTTCATCTTCGTTAACTTGACATCCCCATTTAC | TgStx20 Cas9 plasmid |
| gRNA-TgStx21-R | CTATTTCTAGCTCTAAAACCACTCATCTTGGACGTGACCAACTTGACATCCCCATTTAC | TgStx21 Cas9 plasmid |
| gRNA-TgAAP4-R | TATTTCTAGCTCTAAAACCGGCACTCTTCTTCACTGTTAACTTGACATCCCCATTTA | TgAAP4 Cas9 plasmid |
| gRNA-TgRab11A-R | TATTTCTAGCTCTAAAACCTCTTCACCATGGCGGCTAACAACTTGACATCCCCATTT | TgRab11A Cas9 plasmid |
| TgStx1-AID-F | CGAGCTCGCGCGGCAACTGTCGCCGCAGGCCGCTCCGCGCATGTACCCGTACGACGTC | Fragment used for insertion of 12HA-AID* tag into TgStx1 locus |
| TgStx1-AID-R | CCACGGAGGCGGATCGCGAGTCTCTGAATTTCCACGCATGAGAGCCACCTCCTCCACC |  |
| TgStx20-AID-F | AGTATTGTGTCTTCTCTCTCCCATCTCCTGAACGAAGATGTACCCGTACGACGTCCCC | Fragment used for insertion of 12HA-AID* tag into TgStx20 locus |
| TgStx20-AID-R | AGGGCAGAACCTTCGAGAGGCCGAGAGCCTTGGTCACGTTAGAGCCACCTCCTCCACC |  |
| TgStx21-AID-F | TTCTGGGTTTCAACGCCGGTCAGATACGGTCACGTCCAAGATGTACCCGTACGACGTC | Fragment used for insertion of 12HA-AID* tag into TgStx21 locus |
| TgStx21-AID-R | CTGTGTGGCGAGGCCCATCATCGCAGGTAAGCCCCCCACTAGAGCCACCTCCTCCACC |  |
| TgStx1-Iden-F1 | AAACGGCCTTCTTCGAAGAA | PCR identification of 12HA-AID* insertion in TgStx1 locus |
| TgStx1-Iden-R1 | TTAAATGAGACCGCATGGAA |  |
| AID*-JD-F | CTCCGGCCAAGGCACAA | PCR identification of 12HA-AID* insertion |
| AID*-JD-R | TTTTGGCAGGAAACCAT |  |
| TgStx20-Iden-F | CGAGGAAAGTGTTGTCACTT | PCR identification of 12HA-AID* insertion in TgStx20 locus |
| TgStx20-Iden-R | AGAGGCGTGGAAAAAAGAAG |  |
| TgStx21-Iden-F | GTGGTTCTTCCTCTCGGTAC | PCR identification of 12HA-AID* insertion in TgStx21 locus |
| TgStx21-Iden-R | GTGGTTCTTCCTCTCGGTAC |  |
| EGFP-TgCentrin2-F | TGGACGAGCTGTACAAGATGCAGCGAGGAGCACTGCGA | EGFP-TgHP03- plasmid |
| EGFP-TgCentrin2-R | TAACGTCGTACGGATACATCTACGGGAAAGTCTTCTTGGT |  |
| TgHP03-3MYC-F | GAAGCTTGATGGGGATATCATGGCGTCCTCGGACTCG | TgHP03-3MYC plasmid |
| TgHP03-3MYC-R | TCAGAGATGAGTTTCTGCTCAGCGGAGTCTTGCGGTGG |  |
| TgGT1-3MYC-F | AGAAGCTTGATGGGGATATCATGGCGACGGAGGAGATG | TgGT1-3MYC plasmid |
| TgGT1-3MYC-R | TCAGAGATGAGTTTCTGCTCAACCACCTCCGTCCCCTT |  |
| HA-Stx1-F | ATGCATAGGTAGCCACCATGGCAAGCACAGCACCATCA | Expression of HA-tagged TgStx1 in HEK293T cells |
| HA-Stx1-R | TCAGGAACATCGTATGGGTATTGCCGCATGCTTGACGT |  |
| Flag-Stx20-F | ATGCATAGGTAGCCACCATGAACGTGACCAAGGCTCTC | Expression of Flag-tagged TgStx20 in HEK293T cells |
| Flag-Stx20-F | CCGTCATGGTCTTTGTAGTCCGTCGCGTTGCTCTGCTT |  |
| Flag-Stx21-F | ATGCATAGGTAGCCACCATGAGTGGGGGGCTTACCTGC | Expression of Flag-tagged TgStx21 in HEK293T cells |
| Flag-Stx21-F | CCGTCATGGTCTTTGTAGTCTCGATCGCCTGCCGCATT |  |
| TubDD-EGFP-Rab11a-F | ACGAGCTGTACAAGGATATCATGGCGGCTAAAGAT | EGFP-TgRab11A plasmid |
| TubDD-EGFP-Rab11a-R | TCACTTTCGTCGTAGTCCTAGGCGGAACAGCAGCC |  |
| TgAAP4-3MYC-F | CAAGAATGTCTCTGGGCAGCTCCGGAGTCTTCAGAAACAGGAGCAGAAACTCATCTCT | Fragment used for insertion of 3MYC tag into TgAAP4 locus |
| TgAAP4-3MYC-R | CCCTAATAATCTCGAGTGTCTCCCGCGGCACTCTTCTTCAGATATCCAGATCTTCCTC |  |
| TgAAP4-Iden-F | TACACCTACAAGGTCACTCC | PCR identification of 3MYC insertion in TgAAP4 locus |
| TgAAP4-Iden-R | ATATGCTTATGCATGCCTGG |  |
| TgRab11A-Iden-F | GACGTCTTTCAGACGCTATC | PCR identification of EGFP insertion in TgRab11A locus |
| TgRab11A-Iden-R | CGGCTACATAGAATGTCTCC |  |
